# Interaction between Phage T4 Protein RIII and Host Ribosomal Protein S1 Inhibits Endoribonuclease RegB Activation

**DOI:** 10.3390/ijms23169483

**Published:** 2022-08-22

**Authors:** Augustinas Juškauskas, Aurelija Zajančkauskaitė, Rolandas Meškys, Marija Ger, Algirdas Kaupinis, Mindaugas Valius, Lidija Truncaitė

**Affiliations:** 1 Department of Molecular Microbiology and Biotechnology, Institute of Biochemistry, Life Sciences Center, Vilnius University, Saulėtekio av. 7, LT-10257 Vilnius, Lithuania; 2 Proteomics Centre, Institute of Biochemistry, Life Sciences Center, Vilnius University, Saulėtekio av. 7, LT-10257 Vilnius, Lithuania

**Keywords:** *E. coli* ribosomal protein S1, bacteriophage T4, RIII protein, RNase RegB, lysis inhibition

## Abstract

Lytic viruses of bacteria (bacteriophages, phages) are intracellular parasites that take over hosts’ biosynthetic processes for their propagation. Most of the knowledge on the host hijacking mechanisms has come from the studies of the lytic phage T4, which infects *Escherichia coli*. The integrity of T4 development is achieved by strict control over the host and phage processes and by adjusting them to the changing infection conditions. In this study, using in vitro and in vivo biochemical methods, we detected the direct interaction between the T4 protein RIII and ribosomal protein S1 of the host. Protein RIII is known as a cytoplasmic antiholin, which plays a role in the lysis inhibition function of T4. However, our results show that RIII also acts as a viral effector protein mainly targeting S1 RNA-binding domains that are central for all the activities of this multifunctional protein. We confirm that the S1–RIII interaction prevents the S1-dependent activation of endoribonuclease RegB. In addition, we propose that by modulating the multiple processes mediated by S1, RIII could act as a regulator of all stages of T4 infection including the lysis inhibition state.

## 1. Introduction

Viruses are small pathogens that specifically infect cellular life forms as hosts for their propagation. Due to the limited space of their small genomes, viruses do not encode the complete sets of proteins needed to complete the viral life cycle. Instead, they use resources and employ the biosynthetic functions of the host, so they can survive and propagate only inside the living cells [1,2,3]. Depending on the life cycle and genomic space, some viruses encode enzymes for autonomous genome replication and/or transcription [4,5,6]. However, all known viruses depend on the core components of the host translation machinery to produce viral proteins [1,7,8]. Although new findings indicate that viruses sometimes encode the homologues of ribosomal proteins, these are believed to be acquired from the hosts by a horizontal gene transfer and adapted to control viral protein synthesis in the infected cell [9].

The most striking diversity of virus–host adaptations has been found among the bacteria and their viruses, commonly referred to as bacteriophages or phages [10,11]. Due to their global abundance in the biosphere, both populations evolved a broad spectrum of competitive mechanisms [11,12,13]. The co-evolution of a phage/host system differs depending on the strategy of viral infection, which can vary from strictly lytic (lysing infection) to chronic (constant progeny production without cell lysing), some of which may have an additional temperate state (availability of the prophage state) [14]. Namely the lytic lifestyle in combination with phage diversity has contributed to the diversity of phage–host battle mechanisms [11,15].

Most of the knowledge on host manipulation by large, strictly lytic dsDNA phages has come from the investigation of bacteriophage T4, which infects *Escherichia coli* [16]. The results obtained during more than 70 years of studies on this phage have provided the basis for understanding the lytic life cycle, which typically comprises four stages: (i) host cell entry, (ii) genome replication, (iii) assembly of viral particles, and (iv) pervasion by cell lysis, otherwise known as early, middle, and late periods and cell lysis. The integrity of T4 development is achieved by the temporal control of viral gene expression and the gradual modification of the host proteins. Modification starts from the injection of several phage proteins into the host cell together with T4 genomic DNA [16]. One of these proteins, ADP-ribosyltransferase Alt, ADP-ribosylates the host’s RNA polymerase (RNAP) [17], which is then gradually adapted to transcribe T4 early, middle, and late genes at the correct time [18,19].

The fluent transition from host to phage metabolism is also strongly influenced at the posttranscriptional level. Shutting off the host transcription and translation, as well as degradation of the redundant T4 transcripts, frees up the resources needed for further stages of phage development [20]. Both host- and phage-encoded enzymes are involved in these processes. The T4 early protein RegB endoribonuclease (RNase) cleaves in the middle of the GGAG/U motif preferably located in Shine–Dalgarno (SD) sequences of the translation initiation regions (TIRs) of T4 early mRNAs [21]. These initial cuts inactivate the translation of many early mRNAs and expedite their degradation by the exo- and endo- RNases of the host [20,22]. The functional inactivation of abundant early transcripts that are no longer needed facilitates the transition between the early and later stages of the T4 life cycle. The T4-coded polynucleotide kinase (PNK) [23,24] and the effector protein Srd [25] stimulate the activities of the host endoribonucleases, further decreasing the stability of T4 and host mRNAs. Importantly, depending on the RNA substrate, RegB activity in vitro is stimulated 10- to 100-fold by *E. coli* ribosomal protein S1 [26].

The *E. coli* ribosomal protein S1 is the largest (61 kDa) flexible protein of the ribosome complex [27,28]. Structurally, S1 is composed of six homologous OB-fold domains connected by short linkers [29,30]. Based on biochemical studies, two N-terminal domains (D1 and D2) are required for interaction with the ribosome [31,32], whereas four C-terminal domains (D3-D4-D5-D6) bind various RNAs [29,33,34,35]. In *E. coli*, S1 is an essential protein having multiple activities most of which are related to RNA binding [36,37]. This protein is also known to be recruited by several coliphages for their replication and/or recombination [38,39,40,41,42]. During T4 phage infection, S1 acts as a co-factor of the T4 phage RegB endoribonuclease [26]. S1 binds RNA substrates and promotes cleavage by RegB of the partially structured AG-rich RNAs [43] and unstructured RNA having an 11-nucleotide-conserved sequence [44]. The minimal domain combination required for stimulation of RegB nuclease is D4-D5, whereas the peptide containing all C-terminal domains (D3-D4-D5-D6) stimulates RegB to the same extent as the full-length protein [33,44,45].

The activity of RNase RegB is limited to the early period of T4 infection with no known mechanism of its inactivation. In this study, we provide evidence that the T4-coded protein RIII through direct interaction with the *E. coli* ribosomal protein S1 can weaken RegB activity. Moreover, we propose that the detected S1–RIII interaction may be important for the regulation of many other processes in the infected cell. RIII is quite a small (82 aa) protein, long known to be involved in lysis inhibition (LIN)—a phenomenon characteristic of at least some strictly lytic Tequatroviruses (T2, T4, and T6) [46,47,48]. The LIN process involves several known molecular mechanisms that cause a delay in the infected cell lysis in response to secondary infections by related phages. This helps increase the number of progeny phages produced per infected cell when the number of suitable for infection hosts is limited. Discovered in the middle of the 1950s, LIN is considered to be the first example of virus–virus communications that regulates the nature of virus behavior in response to the virus:host ratio in the environment [49]. RIII protein has been shown to be a cytoplasmic antiholin, which together with the periplasmic antiholin RI, is involved in the regulation of T4 holin-mediated cell lysis [50]. However, it has also been noticed that RIII action in the infected cell could be broader [50,51]. Here, we propose an explanation of how RIII, in concert with the host ribosomal protein S1, could be important for adjusting cell resources between infection stages or in response to changing conditions, such as stress or an increased phage-to-host ratio in the environment. This finding unveils additional layers of the regulation of T4 development and provides the basis for further molecular studies of the processes in the infected cell.

## 2. Results

### 2.1. The Recombinant Protein RIII of T4 Co-Purifies with the Host Ribosomal Protein S1 in Stoichiometric Amounts

**Figure 1:**
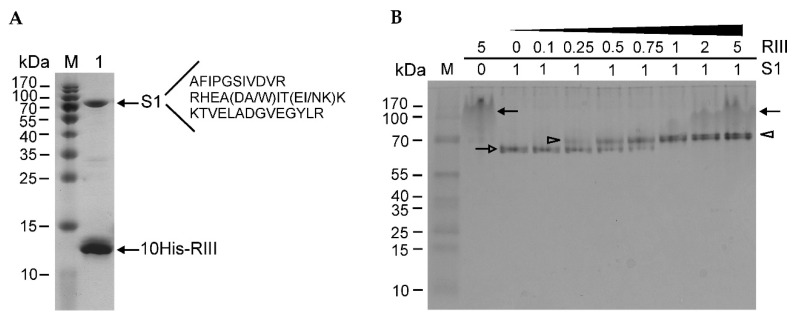
Determination of protein–protein interactions using protein electrophoresis. (**A**) The SDS-PAGE (14%) analysis of the purified N-terminally His-tagged recombinant RIII protein of bacteriophage T4. The amino acid sequences of the identified peptides are listed next to the identified protein. Lanes: M—PageRuler^TM^ Prestained Protein Ladder (Thermo Fisher Scientific, Vilnius, Lithuania); 1—the sample of purified recombinant His-tagged RIII protein. Arrows indicate two co-purified proteins. (**B**) Determination of the stoichiometry of interaction between N-terminally His-tagged RIII protein and *E. coli* ribosomal protein S1. The protein complexes were assembled in vitro and then analyzed by a native PAGE (14%). M—PageRuler™ Prestained Protein Ladder. Numbers above the lanes indicate the molar ratios between RIII and S1. Black arrows show the aggregates of RIII, the open arrow points to the S1 protein, and open arrowheads mark the RIII–S1 complexes.

The N-terminally His-tagged RIII protein of the T4 phage was induced from plasmid p16*rIII*His (Tables S1 and S2) in the *E. coli* expression strain BL21 (DE3) and purified using a standard procedure of His-affinity chromatography. The sodium dodecyl sulfate-polyacrylamide gel electrophoresis (SDS-PAGE) analysis of the purified protein indicated that the recombinant RIII protein was purified together with an unknown host protein having a molecular mass of approximately 70 kDa (Figure 1A).

The band migrating at ~70 kDa was excised and identified using the proteomics methods described in the Materials and Methods section. The results showed that the RIII protein of the T4 phage co-purified together with the ribosomal protein S1 of *E. coli* (Figure 1A and Figure S1). To study protein–protein interactions between the two proteins, we constructed the recombinant plasmids for the overexpression of the N- and C-terminally His-tagged S1 and RIII proteins (Tables S1 and S2). The induced proteins were purified using His-affinity chromatography and dialyzed against the storage buffers. Then, they were examined for the in vitro stoichiometry of protein–protein interactions. For this, the same (constant) molar amounts of S1 protein were mixed with increasing amounts of RIII and the complexes formed between these proteins were analyzed by a native PAGE. The image of the native gel in Figure 1B shows the mobility of the N-terminally His-tagged recombinant proteins RIII and S1 as well as the complexes formed between them. It should be noted that recombinant RIII tends to form protein aggregates that migrate most slowly in the native PAGE. Meanwhile, the mobility of S1 gradually slows down with the increasing amounts of RIII until the ratio of S1:RIII reaches the molar ratio of 1:1 (Figure 1B). As the mobility of the S1–RIII complex then stays at a fixed level in spite of the increasing molar amounts of RIII, we conclude that RIII interacts with S1 at a molar ratio of 1:1.

### 2.2. The RIII Protein of Bacteriophage T4 Mainly Targets the 5th Domain of S1

**Figure 2:**
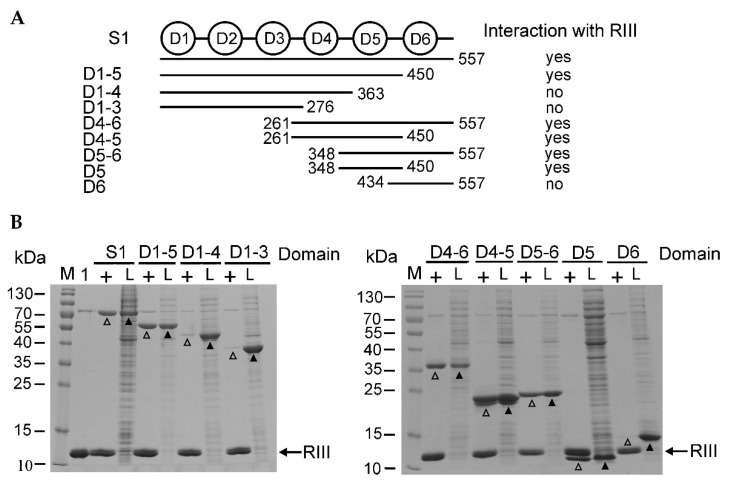
The pull-down assay of in vitro interaction between the recombinant His-tagged RIII protein and untagged S1 as well as its truncated variants. (**A**) The schematic representation of the domains of the intact ribosomal protein S1 (six domains) and its truncated variants used for analysis. The numbers next to the lines mean the amino acid positions of S1. (**B**) The images of the pull-down assay of the induced recombinant proteins followed by SDS-PAGE (14%). The recombinant His-tagged RIII was bound to the His-affinity agarose, which was washed and then mixed with the cell lysates containing either induced intact S1 or its truncated variants (indicated on the top of the image). Lanes: M—PageRuler™ Prestained Protein Ladder (Thermo Fisher Scientific, Vilnius, Lithuania), 1—the sample of the purified His-tagged RIII protein, +—immobilized RIII used as a bait protein, L—the samples of cell lysates having induced prey proteins. The positions of S1 and its truncated variants in lysates are marked with black arrowheads and open arrowheads indicate S1 variants eluted together with the His-tagged RIII. The black arrows show the position of the His-tagged RIII protein.

To determine which S1 domains are the targets for RIII binding, we constructed eight recombinant plasmids for the overexpression of different truncated versions of S1 (Tables S1 and S2). The schematic representation of the domains of the entire protein S1, as well as its truncated versions induced from the expression plasmids, is shown in Figure 2A.

A pull-down assay was used for the examination of in vitro interactions between RIII, S1, and its truncated versions. For this, the His-tagged RIII was immobilized on the His-affinity agarose as a bait protein. Then, the treated agarose gels were washed and the cell lysates containing the induced intact S1 protein or its truncated variants were applied on them. The eluted proteins were analyzed by SDS-PAGE, which showed that all versions of S1 containing the fifth domain were eluted together with the immobilized His-tagged RIII (Figure 2B). The fifth domain alone was sufficient for interaction with RIII (Figure 2B, lane D5+) indicating that this domain of S1 is the main target of RIII. In spite of the trace amounts of non-specifically bound proteins seen in the control experiment (Figure S2B), the amounts of eluted S1 proteins containing the fifth domain were much higher in the presence of immobilized His-tagged RIII (Figure 2B).

### 2.3. The Intact Ribosomal Protein S1 Is the Best Interaction Partner of RIII In Vivo

The Bacterial Adenylate Cyclase Two-Hybrid (BACTH) System [52] was used for the testing of the in vivo interaction between the T4 protein RIII and the *E. coli* ribosomal protein S1 or its truncated variants. For this, the *rpsA* gene coding S1 protein and its truncated variants were fused with the T25 adenylate cyclase fragment in the plasmid pKNT25 (Tables S1 and S2). The *rIII* gene was fused with the T18 adenylate cyclase fragment in the plasmid pUT18 (Tables S1 and S2). We also wished to test the possible in vivo interaction between the ribosomal protein S1 and the T4 RegB endoribonuclease, which has a dependency on the S1 protein for its activity [26]. Therefore, the T18 fragment was also fused with the T4 gene *regB.* The assay was performed according to the protocol of the BACTH system kit as described in the Materials and Methods section.

**Figure 3:**
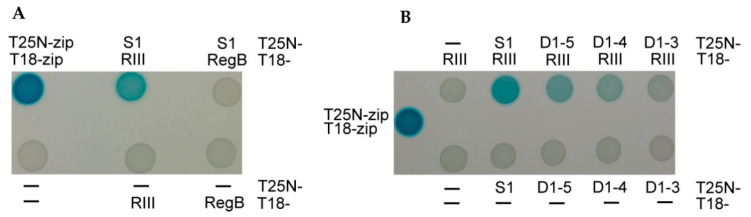
Analysis of in vivo protein–protein interactions of the ribosomal protein S1 with the T4 proteins RegB and RIII using the BACTH system [52]. (**A**) Interactions between the intact proteins S1 and RIII, and S1 and RegB. (**B**) Interactions of RIII protein with the intact protein S1 or its truncated variants. Positive control—T18 and T25 fragments fused with leucine zippers; negative control—T18 and T25 adenylate cyclase fragments without fusions (marked by **–**); T25N and T18 fusions with different proteins are specified next to the spot results. Blue color of the spots indicates adenylate cyclase activity due to the interaction between proteins fused to the T18 and T25 fragments.

As expected, no protein–protein interaction was observed (white spots) when combining the plasmids encoding the adenylate cyclase fusions with RegB and S1 (Figure 3A). This confirmed the previously proposed hypothesis that the activation of RegB nuclease by S1 does not depend on direct interaction between these proteins [53]. Meanwhile, this assay confirmed an in vivo RIII–S1 interaction (blue spots) (Figure 3).

The interaction of RIII with the truncated S1 proteins weakened with the decreasing number of S1 domains fused (Figure 3B). These results indicate that the intact ribosomal protein S1 is the best partner for the interaction with the RIII protein of the T4 phage in vivo.

### 2.4. The Protein RIII Inhibits Endoribonuclease RegB Activation by S1

As already mentioned, the activity of the T4 RegB endoribonuclease toward T4 early mRNAs depends on activation by the ribosomal protein S1 of the host. Namely the D4 and D5 domains of S1 are necessary for RegB activation [45]. Since RIII also interacts with S1 variants containing D5, we decided to test the influence of RIII on the ability of S1 to activate the RegB nuclease. For this, we purified the His-tagged proteins S1, RIII, and RegB (Figure S3B) and performed the in vitro RegB cleavage reactions using the total RNA isolated from *E. coli* cells infected by the T4 ∆*rIII*∆*regB* double mutant constructed in this study (Figure S3A).

**Figure 4:**
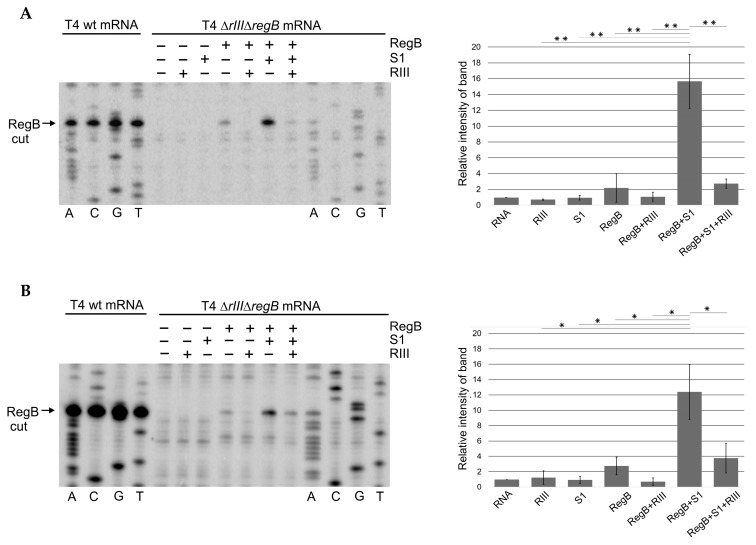
Primer extension analysis of T4 early mRNAs in vitro exposed to the diverse sets of recombinant proteins. (**A**) The representative image and analysis of RegB cleavage using 5′-^32^P-labeled primer No. 35 complementary to the gene *motA* mRNA. (**B**) The representative image and analysis of RegB cleavage using 5′-^32^P-labeled primer No. 36 complementary to the gene *30.7* mRNA. Total RNA was isolated from the *E. coli* cells 2 min post-infection with either phage T4 wt or T4 ∆*rIII*∆*regB* at 37 °C. The 20 μg aliquots of RNA were subjected to either Sanger dideoxy sequencing or primer extension analysis. The latter analysis was performed after exposure of RNA to different sets of the His-tagged recombinant proteins specified at the top of the lines. The quantity of RegB-cleaved mRNAs was analyzed using GelAnalyzer and Microsoft Excel as described in the Methods section. All values are means ± SD from three independent experiments (Figure S4) (* *p* < 0.03, ** *p* < 0.02, Welch’s *t*-test).

The total RNA isolated from *E. coli* cells infected with T4 ∆*rIII*∆*regB* was used as a source of RegB substrates in seven in vitro reaction mixtures as follows: (i) only total RNA in the reaction buffer; (ii) RNA + RIII; (iii) RNA + S1; (iv) RNA + RegB; (v) RNA + RegB + RIII; (vi) RNA + RegB + S1; and (vii) RNA + RegB + S1 + RIII. The products of these reactions were then analyzed by primer extension using the 5′-P^32^-labeled primers No. 35 and No. 36 (Table S1), complementary to the T4 early transcripts *motA* [54] and *30.7* [55], respectively, as they were known to have RegB cleavage sites. The representative images showing RegB activity toward the T4 ∆*rIII*∆*regB* early transcripts *motA* (A) and *30.7* (B) in different mixtures are presented in Figure 4.

The densitometric analysis of the bands resulting from the RegB cleavage at its specific site GGAG present in these transcripts confirmed that RegB activity was indeed activated by S1, but this activation was abolished by the RIII protein added to the reaction mixture containing mRNA, RegB, and S1 (Figure 4).

## 3. Discussion

The bacteriophage T4 and *E. coli* laboratory strains represent one of the most studied phage/host systems and answer many questions about the strictly lytic life cycle of large dsDNA phages. Due to the availability of the mutant collections and expression systems of the host, T4 continues to be one of the most explored phages in basic and applied research. This has allowed researchers to reveal new details about already known mechanisms and find novel phage–host interactions still hidden in the large genome of this phage. In this study, we also provide evidence of the previously unknown protein–protein interactions between the host ribosomal protein S1 and T4-encoded protein RIII. Historically, the *rIII* gene was known from the rapid lysis (r) mutants of T4 having a large, sharp-edged phenotype of plaques compared to the small plaques of T4 wt [47,48,56,57]. Here, we show that the T4 recombinant RIII protein co-purifies with the host protein S1 at the stoichiometric amounts (Figure 1). The pull-down assays show that in vitro RIII preferably interacts with the domain D5 of the S1 protein (Figure 2). Meanwhile, the results obtained using the *E. coli* BACTH system [52] indicate that in vivo, the strongest interaction is seen between the intact proteins (Figure 3).

The *E. coli* ribosomal protein S1 is a multifunctional protein composed of six homologous RNA-binding domains that bind very different RNAs [37]. All known functions of S1 are related to RNA binding but different S1 domains are involved. The first two N-terminal domains (D1 and D2) are involved in binding to the ribosome complex [31] and the interaction with the Qβ phage replicase [58]. The second of them (D2) is required for binding to tmRNA [59]. The C-terminal domains (especially D3 to D5) bind various mRNAs [33,34,35,36,40,60]. Based on the structural data, the D4–D6 domains may be implicated in ribosome inactivation under stress conditions [61]. Domains D5 and D6 help stimulate transcription [62,63,64], whereas D6 is specifically involved in S1 autogenous control [65]. The T4 phage has adapted to take advantage of the main function of S1—translation initiation—to stimulate the activity of the viral RNase RegB. The C-terminal domains from D3 to D6 of S1 accelerate a RegB-dependent cleavage within the SD sequence of T4 early mRNAs when their translation is no longer required [26,66]. Since direct interactions between RegB and S1 have not been detected and it was noticed that RegB has only a low affinity for its RNA substrates, it was proposed that S1 stabilizes the S1–mRNA–RegB complex via a primary step of mRNA binding [53].

In this study, we confirm that in vivo RegB and S1 proteins do not interact directly, as it was predicted previously [53]. Meanwhile, we detected that S1 interacted with the T4 protein RIII and this interaction prevented RNase RegB from activation by S1 in vitro (Figure 4 and Figure S4). Since the stimulation of RegB is thought to be mediated by the RNA-binding function of S1, we speculate that RIII binding prevents the C-terminal domains of S1 from mRNA binding. A similar effect has been observed in the *Pseudomonas aeruginosa* giant phage phiKZ, where the phage-encoded effector protein Dip occluded the RNA-binding sites of the host RNase E leading to the inhibition of its activity [67]. Although the regulation of the host ribosomal proteins by viral factors is common between viruses [8], to our knowledge, RIII is the first identified effector protein of T4 targeting S1 and the first one affecting ribosomal proteins at all. Taking into account that S1 in *E. coli* plays many different roles and binds very different RNAs, the function of the S1–RIII interaction in T4-infected cells may not be restricted to the functional inactivation or regulation of RegB. In fact, RIII may modulate the expression regulation of many host and phage genes. To better understand all the possible outcomes of the S1–RIII interaction, we would like to briefly comment on the key points of the T4 life cycle that may be targeted by this interaction.

When T4 infects *E. coli*, its life cycle inside the host cell under optimal laboratory conditions lasts about 25 min. The process involves the timely expression of about 300 genes, whose transcription is regulated by three classes of promoters: early, middle, and late [18,19,68]. The products of early genes are thought to usurp host functions and fight hosts’ anti-viral defenses, whereas those expressed in the middle and late periods are mainly required for the replication and production of virions, respectively. The infection cycle ends by cell lysis when T4 holin polymerizes to form holes in the cytoplasmic membrane of the infected cell leading to its lethal permeability [69]. However, T4 replication inside the infected cell can be prolonged for hours in response to superinfection by T4 or other Tequatroviruses after 5 min of the primary infection. This phenomenon, called LIN, leads to the delay of cell lysis until the burst size of T4 substantially increases. The processes leading to the LIN state involve a signal carried by the virions of the superinfecting phages, which induces holin stabilization to prevent the formation of holes [69]. Two antiholins, the periplasmic RI and cytoplasmic RIII, interact with the soluble domains of holin and directly stabilize the LIN state [50,69,70,71]. However, the LIN process involves many other T4 factors that act in the signal transduction, adjusting of the intracellular environment, or under certain conditions [49,72,73]. In principle, through interaction with S1, RIII may control all these processes.

The expression of the *rIII* gene in T4 is regulated by all types of promoters, early (the most proximal), middle, and late [68]. Accordingly, RIII protein in infected cells at 30 °C is detected after 5 min of infection and reaches a plateau at about 35 min after infection [74]. This is consistent with the requirement of RIII as an antiholin factor but also indicates that the RIII protein may be important during the entire T4 life cycle and possibly plays other functions. Through interaction with the C-terminal domains of S1, RIII may modulate all functions performed by this protein, including transcription, RNA processing, and translation of the host and/or phage genes. For example, RIII may be at least in part responsible for the shutting off of host transcription and translation—the processes that start during the early period of infection [16]. Also, it may control the protein synthesis of T4, as this phage, like all other viruses, relies on host ribosomes for the translation of its mRNAs.

The mechanisms by which T4 competes with the host for its translation apparatus are not well understood. The differential abundance and specific structure of T4 transcripts with slightly stronger TIRs may favor the translation of certain T4 mRNAs [68,75]. Furthermore, like many large lytic dsDNA phages, T4 encodes its own tRNAs that are thought to optimize T4 protein synthesis [76]. However, little is still known about the influence of the T4-encoded protein effectors or the chemical modifications that may enable the phage to manipulate host ribosomal proteins and/or translation factors. It has been found that some of them, including the proteins S1 and EF-Tu, undergo ADP-ribosylation by the T4 ADP-ribosyltransferases Alt and ModB, respectively [77,78]. The consequences of this modification are unexplored, but certainly, it may be important for the modulation of the S1–RIII interaction.

Translation initiation is a key, in most cases the rate-limiting step of protein synthesis, which is often targeted by viruses [1,7,79]. The TIRs of prokaryotic mRNAs are recognized by the ribosomal 30S subunit through 16S ribosomal RNA base-pairing with an SD sequence proximal to the AUG start codon. The competitive interactions between TIR-binding proteins commonly modulate translation initiation in *E. coli* and its phages. For example, some T4 proteins act as autogenous translational repressors [20]. The ribosomal protein S1 can also be considered a TIR-binding protein so its modification by RIII binding may also modulate the initiation of the translation of many *E. coli* and/or T4 mRNAs.

Finally, translated mRNAs are usually more stable, as ribosomes occlude target sites for endoribonucleases [80,81]. Therefore, by the modulation of the translation efficiency of certain mRNAs, the S1–RIII complex may also modulate their stability. In this work, we show that RIII abolishes the in vitro activation of RNase RegB by S1, thereby slowing the cleavage of known RegB targets located in the SD regions of T4 early transcripts *motA* [54] and *30.7* [55]. By cleaving the SD sequences of early mRNAs, RegB blocks their translation and expedites degradation by the endo- and exo-RNases of the host [23]. In this context, RIII may also play a role in the modulation of the stability of T4 and/or the host mRNAs.

**Figure 5:**
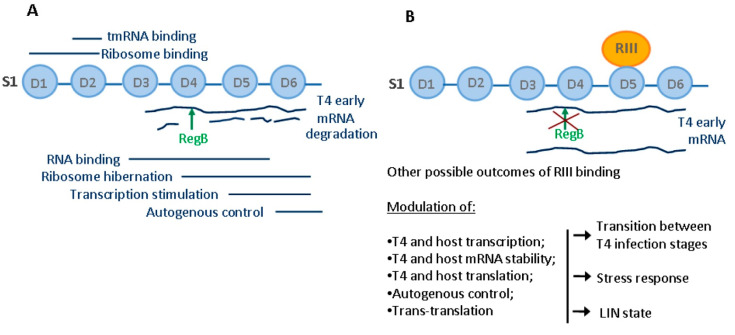
Functions of the ribosomal protein S1 in *E. coli* and possible outcomes of RIII–S1 interaction. (**A**) Known functions of S1 domains in *E. coli*. (**B**) Possible outcomes of RIII binding to S1 during T4 infection.

To conclude, in this study, we identified a unique interaction between *E. coli* ribosomal protein S1 and T4-coded protein RIII, which was previously known to be involved in holin stabilization. Here, we propose that due to the multifunctional nature of S1, RIII may be a global modulator of intracellular processes in T4-infected cells. Through binding to S1, RIII may interfere with all biological activities of this ribosomal protein leading to the regulation of multiple processes of the infected cell (Figure 5).

We show that RIII indeed abolishes RegB activation by S1 so it may affect the expression of at least those genes that have RegB processing sites. Moreover, the expression profile of RIII indicates that RIII may contribute to the correct balance of intracellular resources during all stages of viral infection including the LIN state. The ADP-ribosylation of S1 that occurs early in infection may also be important, but our results show that RIII acts as a viral effector protein targeting mainly the RNA-binding function, which is central for all S1 activities. By modulating multiple processes mediated by S1, RIII may contribute to the correct balance of intracellular resources during all stages of T4 infection.

## 4. Materials and Methods

### 4.1. Escherichia coli Strains, Phages, and Plasmid Vectors

The *E. coli* strains, bacteriophages, and plasmid vectors used in this study are listed in Table 1.

Bacterial strains and phages were grown at 37 °C in a standard LB medium (NaCl 10 g L^–1^, tryptone, 10 g L^–1^, and yeast extract 10 g L^–1^, adjusted to pH 7.0 with NaOH) and LB agar (LB medium with 15 g L^–1^ agar) unless otherwise stated. When required, the growth media were supplemented with an appropriate antibiotic (ampicillin 50 μg mL^–1^ or 100 μg mL^–1^, kanamycin 50 μg mL^–1^; streptomycin 100 μg mL^–1^) or specified amounts of 5-bromo-4-chloro 3-indolyl-β-D-galactopyranoside (X-gal) and isopropyl-β-D-thiogalactoside (IPTG). For the preparation of the M9 minimal medium, the 1.5% M9-agar, 5 × M9 salts, 20% glucose, 1 M MgSO_4_, and 0.1 M CaCl_2_ were prepared separately; 5 × M9 salts: Na_2_HPO_4_ × 7H_2_O—64 g L^–1^, KH_2_PO_4_—15 g L^–1^, NaCl—2.5 g L^–1^, and NH_4_Cl—5.0 g L^–1^.

### 4.2. PCR and DNA Cloning

The enzymes used for PCR and DNA cloning were purchased from Thermo Fisher Scientific (Vilnius, Lithuania). The T4 genes to be cloned were PCR amplified directly from phage plaques using a Phusion High-Fidelity PCR Master Mix (2×). The plasmid DNA isolation and purification of DNA fragments were performed using commercial spin column kits GeneJET Plasmid Miniprep Kit and GeneJET Gel Extraction and DNA Cleanup Micro Kit, respectively. A DreamTaq PCR Master Mix (2×) was used for test PCRs. The cloned genes were sequenced at Macrogen (Maastricht, The Netherlands). The oligonucleotide primers used for PCR reactions were purchased from either Metabion (Planegg/Steinkirchen, Germany) or Thermo Fisher Scientific (Vilnius, Lithuania). The sequences of DNA primers used in this study are listed in Table S1 and the details of the plasmid construction are presented in Table S2.

### 4.3. Overproduction and Purification of the Recombinant Proteins

*E. coli* BL21 (DE3) (Novagen, Madison, WI, USA) cells harboring expression plasmids with the inserted gene were grown individually at 37 °C in an LB medium supplemented with ampicillin (50 μg mL^–1^) until the OD_600_ reached 0.6. The expression was induced by adding IPTG to a final concentration of 0.1 mM, and growth continued for 18 h at 20 °C. To obtain larger amounts of recombinant RegB, the *regB* gene was cloned by introducing mutations in two RegB targets GGAG present in the SD sequence and coding sequence (Tables S1 and S2). For the overexpression of this gene, the cells were grown until the OD_600_ reached 0.9, the final concentration of IPTG was 1 mM, and the time of induction was 30 min at 37 °C. The cells were then harvested by centrifugation (20 min, 4000× *g* at 4 °C), suspended in a buffer (50 mM sodium phosphate, pH 7.7, 300 mM NaCl, 10 mM imidazole, 0.03% Triton X-100), and disrupted by sonication (Bandelin Sonopuls, Berlin, Germany). Afterward, cell extracts were centrifuged for 20 min at 14,000× *g* at 4 °C and the samples were subjected to a 14% SDS-PAGE analysis. The resulting protein bands were visualized by staining with a Coomassie Brilliant Blue G-250 solution (Thermo Fisher Scientific, Vilnius, Lithuania). The soluble His-tagged proteins were purified using a His-Spin Protein Miniprep^TM^ kit (Zymo Research, Irvine, CA, USA) and used for either SDS-PAGE analysis or in vitro experiments. The eluted fraction of recombinant proteins RIII and S1 were dialyzed against a 100-fold volume of Tris HCl 50 mM pH 7.4, 100 mM NaCl, and 10% glycerol to remove imidazole. The eluted fraction of RegB was dialyzed against a 100-fold volume of Tris HCl 50 mM pH 7.4, 100 mM NaCl, 1 mM DTT, 0.1 mM EDTA, and 10% glycerol. The concentration of the proteins in the storage solutions was determined using the Bradford reagent (Thermo Fisher Scientific, Vilnius, Lithuania) according to the manufacturer’s protocol, and molar concentrations were calculated.

### 4.4. Proteomics Analysis

The protein bands of interest were excised from Coomassie Brilliant Blue R-250 (Biorad, Hercules, CA, USA)-stained SDS-PAGE gel and destained two times using 200 μL of 25 mM ammonium bicarbonate in 50% acetonitrile for 30 min at 37 °C. The samples were reduced before trypsin digestion with 50 μL of 50 mM TCEP in 25 mM ammonium bicarbonate (pH 8.0) for 10 min at 60 °C and alkylated with 100 mM iodoacetamide in 25 mM ammonium bicarbonate at room temperature in the dark for 1 h. The gel bands were desiccated with 100 μL of acetonitrile, air-dried, and reconstituted with 40 µL of 10 ng µL^–1^ TPCK trypsin (Thermo Fisher Scientific, Vilnius, Lithuania). The samples were digested at 37 °C for 18 h. The tryptic digest peptides were extracted from the gels two times with 50 µL of 5% trifluoroacetic acid in 50% acetonitrile at 37 °C for 1 h. The extracts were dried in a concentrator (Eppendorf, Hamburg, Germany), reconstituted with 10 µL of 0.1% trifluoroacetic acid, and desalted using ZipTip Pipette Tips (Merck Millipore, Darmstadt, Germany) following the manufacturer’s instructions.

De novo sequencing of the tryptic digest peptides was carried out on a 4800 MALDI TOF/TOF mass spectrometer (Applied Biosystems/MDS SCIEX, Waltham, MA, USA). An amount of 0.5 μL of the sample on a 384 spot Opti-TOF sample plate was overlayed with 0.5 μL of 4 mg mL^–1^ α-cyano-4-hydroxycinnamic acid (Merck, Darmstadt, Germany) in 50% acetonitrile. The peptide mass spectra were acquired in MS reflector positive mode in the 800–4000 mass range. The dominant peaks were subsequently fragmented in MS/MS positive mode and 1 kV collision energy without CID gas or CID was applied with medium air pressure. GPS Explorer De Novo Explorer software was used for the peptide sequence generation. Data reproduced in at least three independent MS/MS runs were combined to obtain the final peptide sequences. The BLASTp analysis of the peptide sequences obtained was used for the identification of their source protein.

### 4.5. Pull-Down Assays

An *E. coli* BL21 (DE3) (Novagen, Madison, WI, USA) strain was transformed with the plasmids pET21d (Novagen, Madison, WI, USA), p21d*rIII*His, p21*rpsA*, p21*rpsA*-D1-3, p21*rpsA*-D1-4, p21*rpsA*-D1-5, p21*rpsA*-D4-5, p21*rpsA*-D4-6, p21*rpsA*-D5-6, p21*rpsA*-D5-5, and p21*rpsA*-D6-6 (Tables S1 and S2). The induction of the protein expression, centrifugation, and disruption of the induced cells were carried out as described above. The pull-down assays were performed using the His-Spin Protein Miniprep^TM^ kit (Zymo Research, Irvine, CA, USA). For this, the N-terminally 6His-tagged RIII protein from the lysates of induced *E. coli* BL21 (DE3) cells was immobilized onto nickel-charged His-affinity agarose. The unbound proteins were washed out with the washing buffer of the kit (50 mM sodium phosphate buffer, pH 7.7, 300 mM NaCl, 50 mM imidazole, 0.03% Triton X-100), and then cell lysates containing induced, intact S1 or its truncated variants were applied on the same agarose. After incubation at room temperature for 10 min, the unbound proteins were washed twice with the washing buffer of the kit. The protein complexes formed between the immobilized 6His-tagged RIII protein of the T4 phage and the recombinant variants of the S1 protein were eluted using the imidazole-containing elution buffer of the kit (50 mM sodium phosphate buffer, pH 7.7; 300 mM NaCl, 250 mM imidazole). The samples of eluted proteins were then directly subjected to a 14% SDS-PAGE and the proteins were visualized by Coomassie blue staining, as described above. For the negative control experiments, the soluble fraction of induced *E. coli* BL21 (DE3) cells harboring plasmid vector pET21d was poured onto nickel-charged His-affinity agarose and the agarose was washed with the washing buffer of the kit. Then, the cell lysates containing induced, intact S1 protein or its truncated variants were applied on the same agarose, incubated for 10 min, and washed twice with the washing buffer. The protein complexes were eluted using the imidazole-containing elution buffer of the kit and analyzed as described above.

### 4.6. Determination of Interaction Stoichiometry

In order to determine the stoichiometry of the interaction between the S1 and RIII proteins, the *E. coli* BL21 (DE3) (Novagen, Madison, WI, USA) was transformed with the plasmids p16*rpsA*His and p21d*rIII*His (Tables S1 and S2) and grown to OD_600_ ≈ 0.6 in 30 mL of LB medium containing 50 μg mL^–1^ of ampicillin. The induction of proteins and the purification and dialysis of the eluted proteins were carried out as described above. The S1 and RIII proteins were purified from the induced cells as described, and the eluted proteins were dialyzed overnight against a 100-fold volume of a 50 mM sodium phosphate, pH 7.4, and 100 mM NaCl buffer to remove imidazole. The concentration of proteins in the storage solutions was determined using the Bradford reagent (Thermo Fisher Scientific, Vilnius, Lithuania) according to the manufacturer’s protocol, and molar concentrations were calculated. Afterward, protein solutions were mixed in different molar ratios (RIII:S1—0.1:1; 0.25:1; 0.5:1; 0.75:1; 1:1; 2:1, and 5:1) and incubated at room temperature for 10 min to facilitate the in vitro formation of the S1–RIII complex. Then, an SDS-free loading sample buffer was added to each sample including the pure RIII and S1 controls, and the samples were analyzed by a native 14% PAGE. The resulting protein bands were visualized by staining with a Coomassie Brilliant Blue G-250 solution (Thermo Fisher Scientific, Vilnius, Lithuania).

### 4.7. Assay of Protein Interactions in a Bacterial Two-Hybrid System

The in vivo protein–protein interactions of the T4 proteins RIII and RegB with the intact S1 protein or its truncated variants were assayed using the Bacterial Adenylate Cyclase Two-Hybrid (BACTH) System kit (Euromedex, Souffelweyersheim, France). For this, the *rpsA* gene, as well as its truncated variants, was inserted into the pKNT25 plasmid (Table 1) between the XbaI and SacI sites yielding the pKNT25S series of the plasmids (Tables S1 and S2). The *rIII* and *regB* genes were inserted into the pUT18 plasmid (Table 1) between the XbaI and KpnI sites (Tables S1 and S2). In this way, the *rpsA* gene and its truncated variants were fused with the T25 adenylate cyclase fragment and *rIII* and *regB* were fused with the T18 adenylate cyclase fragment. Two plasmids of the kit pKT25-zip and pUT18C-zip (Table 1) were used as a combination for the positive control. Different pairs of plasmids were co-transformed into BTH101 cells and grown overnight at 30 °C in an LB medium containing appropriate antibiotics (100 μg mL^–1^ ampicillin, 50 μg mL^–1^ kanamycin). For fresh culture, 2 mL of the LB medium with the aforementioned antibiotics was inoculated with 20 μL of overnight culture and grown to OD_600_ ≈ 0.6. For the plate assay, 2 μL of cell cultures were spotted on 1 × M9 minimal medium agar plates supplemented with 0.2% glucose, 2 mM MgSO_4_, 0.1 mM CaCl_2_, 0.5 mM IPTG, 60 μg mL^–1^ X-Gal, 100 μg mL^–1^ ampicillin, and 50 μg mL^–1^ kanamycin and incubated at 30 °C for 48 h in order to determine the protein interactions expressed by the blue color of the spots.

### 4.8. Construction of T4 Phage Mutants

The deletions to be introduced into the genome of the T4 phage were first constructed on the basis of plasmid vectors pBSPLO+ [82] or pJet1.2 (Thermo Fisher Scientific, Vilnius, Lithuania). The details of the plasmid construction are given in Table S2. For the construction of T4 ∆*rIII* (Table 1, Figure S3), the recombinant plasmid pT4*rIII*del carrying the deletion between the cloned DNA fragments flanking the *rIII* gene was used for the transformation of *E. coli* DH10B (Thermo Fisher Scientific, Vilnius, Lithuania) under selectable conditions (ampicillin, 50 μg mL^–1^). The transformed cultures were grown in an LB medium supplemented with ampicillin at 37 °C to OD_600_ ≈ 0.6 and infected with T4 wt phage at a multiplicity of infection (MOI) of 3 followed by its propagation until lysis of the culture. Phage propagation in the presence of replicating plasmid carrying T4-specific sequences led to the homologous recombination between plasmid and the T4 genome resulting in a mix of progeny phages. The diluted lysates were then plated using double-layer LB agar plates and incubated at 37 °C overnight. The T4 ∆*rIII* mutant was selected according to the characteristic plaque phenotype (larger, sharp-edged, clear plaques) and verified by PCR and sequencing with the specific primers No. 27 and No. 28 (Table S1).

For the construction of the double mutant T4 ∆*rIII*∆*regB* (Table 1, Figure S3) the recombinant plasmid pT4*regB*del carrying the *E. coli lacZ alpha* fragment inserted between T4 *regB* gene-flanking sequences was used for the transformation of *E. coli* DH10B under selectable conditions (ampicillin 50 μg mL^–1^). The transformed culture was grown as described above and infected with the T4 ∆*rIII* mutant (MOI = 3). The resulting lysates of the culture were then serially diluted and plated using double-layer LB agar plates in the presence of X-gal (200 μg mL^–1^) and IPTG (0.5 mM). After overnight incubation, the T4 ∆*rIII*∆*regB* mutant was selected as large, blue-edged plaques (Figure S3A) and verified by PCR and sequencing with the specific primers No. 29 and No. 34 (Table S1).

### 4.9. Isolation of Total RNA from Infected Cells

The total RNA from phage-infected *E. coli* cells was phenol extracted as described [21]. For this, *E. coli* BTH101 liquid cultures were grown in an LB medium supplemented with streptomycin (50 μg mL^–1^) in two separate flasks at 37 °C with shaking (180 rpm). When the cultures reached OD_600_ ≈ 0.8, the T4 wt and T4 ∆*rIII*∆*regB* phages were added to the separate flasks at an MOI = 10. To isolate a total RNA having T4 early transcripts, the aliquots of 3 mL of infected cultures were taken 2 min after infection and immediately mixed with 3 mL of boiling lysis buffer (SDS 2%, EDTA 4 mM). The mixture was then boiled for 5 min and used for phenol extraction of the total RNA. The extracted RNA was precipitated by adding a 1/10 part of 5 M NaCl and 2.5 parts of 96% ethanol. The total RNA was collected by centrifugation and the pellet was suspended in 20 μL of nuclease-free water. The prepared RNA was stored at −20 °C until used for sequencing or in vitro analysis of RegB nuclease activity.

### 4.10. In Vitro Activity Tests of RegB Endoribonuclease

The in vitro activity of the RegB endoribonuclease of the T4 phage was tested using recombinant proteins produced as described above. The total RNA isolated from phage-infected cells was used as a source of the RegB substrates. The recombinant protein S1 was used as an activator of RegB activity, and RIII protein was tested for its role during S1-activated RegB cleavage. Seven 20 μL reaction mixtures were prepared each containing 20 μg of total RNA in a reaction buffer of RegB (50 mM Tris-HCl pH 7.4, 1 mM DTT and 0.1 mM EDTA) and different sets of recombinant proteins, as follows: (i) only total RNA in the reaction buffer; (ii) RNA + RIII; (iii) RNA + S1; (iv) RNA + RegB; (v) RNA + RegB + RIII; (vi) RNA + RegB +S1; and (vii) RNA + RegB +S1 + RIII. The molar ratio of RegB:S1 was 1:2 and that of S1:RIII was 1:3. The reaction mixtures were incubated at 37 °C for 5 min and stopped by directly adding the deproteinization suspension containing 100 μL of phenol:chloroform (1:1) and 80 μL H_2_O. After deproteinization, RNA from the reaction mixtures was precipitated by adding a 1/10 volume part of 5M NaCl and 2.5 parts of 96% ethanol at −20 °C for at least 2 h. Then, RNA was collected by centrifugation at 16,000× *g* for 15 min and washed with 70% ethanol. When ethanol was removed, the RNA was suspended in the reaction buffer of an AMV RT (Promega, Madison, WI, USA) and used for primer extension analysis.

### 4.11. Primer Extension Analysis of T4 mRNA

A total of 50 μg of RNA isolated from T4-infected *E. coli* cells was used for dideoxy chain termination sequencing [84] of T4 mRNAs using Avian Myeloblastosis Virus (AMV) Reverse Transcriptase (Promega, Madison, WI, USA) and specific primers No. 35 and No. 36 (Table S1). The primers were 5′-end-labeled using ATP (γ-^32^P) (Perkin Elmer, Boston, MA, USA) and T4 PNK (Thermo Fisher Scientific, Vilnius, Lithuania). The annealing of 2 pmol of the labeled primer to 50 μg of RNA in a volume of 10 μL was performed at 60 °C followed by cooling on ice. Then, the AMV reverse transcriptase (5 units) was added and four chain-termination reaction mixtures, each containing 2.5 μL of the annealing mixture and 2.5 μL of a solution containing all four dNTPs (2 mM each) and one of the four dideoxynucleotides (1 mM), were set. The mixtures were incubated for 20 min at 48 °C. The reaction was stopped by adding 6 μL of stop solution (formamide 95%, 20 mM EDTA pH 8.0, bromophenol blue 2%, Xylene cyanol FF 2%). Primer extension analysis of the RNAs that were subjected to in vitro activity tests of RegB nuclease was performed using the same labeled primers (1 pmol per reaction) but the reactions were performed using all treated RNAs (each ~20 μg) in the presence of 1mM dNTP only. The products of all reactions were analyzed by electrophoresis on a 6% denaturing polyacrylamide gel (8 M urea, TBE) and visualized using a Fujifilm FLA-5100 phosphorimager (Tokyo, Japan).

### 4.12. Densitometric Analysis of Gel Images

The intensity of the RegB cut representing the band in the primer extension gel images was analyzed using GelAnalyzer version 19.1 [85]. The bands were initially detected automatically and the bands corresponding to the mRNA cut by RegB were normalized manually to the same width in pixels according to the lane with the widest detected bands. The background was subtracted automatically using the morphological method provided in the program with a 50% peak width tolerance. The total volume of the peak was calculated using the software and the relative intensities of the bands were calculated with the intensity of the band in the protein-untreated sample taken as 1. Microsoft Excel functions AVERAGE and STDEV.P were used to determine the average and standard deviations of the relative band intensity, respectively, among the three independent experiments. A one-tailed Welch’s *t*-test was used to identify significant differences between treatment groups using the Microsoft Excel function T.TEST.

## Figures and Tables

**Table 1:** *Escherichia coli* strains, phages, and plasmid vectors used.

*E. coli* Strain	Relevant Characteristics	Used for	Source/Reference
DH10B	F^–^ *endA1 recA1 galE15 galK16 nupG rpsL ΔlacX74 Φ80lacZΔM15 araD139 Δ(ara,leu)7697 mcrA Δ(mrr-hsdRMS-mcrBC) λ^-^*	Cloning, construction of T4 mutant	Thermo Fisher Scientific
BL21 (DE3)	*E. coli* B F^–^ *dcm ompT hsdS(*rB^–^ mB^–^*) gal* λ(DE3)	Superexpression of cloned genes	Novagen
BTH101	F^–^ *cys-99 araD139 galE15 galK16 rpsL1 (Str^R^) hsdR2 mcrA1 mcrB1*	Experiments in BACTH system; T4 propagation; isolation of total RNA from infected cells	Euromedex/[52]
**Phage**			
T4 wt	Wild-type (T4D+)	Infection of *E. coli*	A gift from Dr. W.B. Wood
T4 ∆*rIII*	T4 wt carrying deletion of gene *rIII*.	Infection of *E. coli*	This study
T4 ∆*rIII*∆*regB*	T4 wt carrying deletions of genes *rIII and regB*.	Infection of *E. coli*	This study
**Plasmid**			
pET21a	Ap^R^; expression vector encoding IPTG-inducible T7 promoter	Cloning	Novagen
pET21d	Ap^R^; expression vector encoding IPTG-inducible T7 promoter	Cloning	Novagen
pET28a	Km^R^; expression vector encoding IPTG-inducible T7 promoter	Cloning	Novagen
pET16b	Ap^R^; expression vector encoding IPTG-inducible T7 promoter	Cloning	Novagen
pKNT25	Km^R^; expressing T25 fragment (first 224 aa of CyaA) under control of a lac promoter.	Cloning and negative control of protein–protein interaction	Euromedex/[52]
pUT18	Ap^R^; expressing T18 fragment (225 to 399 aa of CyaA) under control of a lac promoter.	Cloning and negative control of protein–protein interaction	Euromedex/[52]
pKT25-zip	Km^R^; expressing the leucine zipper of GCN4 fused to the T25 fragment under control of the lac promoter.	Positive control of protein–protein interaction	Euromedex/[52]
pUT18C-zip	Ap^R^; expressing the leucine zipper of GCN4 fused to the T18 fragment under control of the lac promoter.	Positive control of protein–protein interaction	Euromedex/[52]
pJet1.2 blunt	Ap^R^; blunt Cloning vector	Cloning for recombination	Thermo Fisher Scientific
pBSPLO+	Ap^R^; vector designed for T4 homologous recombination	Cloning for recombination	A gift from Dr. K.N. Kreuzer [82]
pRA6-2	carries the 0.43-kb HpaI-BglII T4 DNA fragment with gene *31*	DNA fragment for cloning	[83]

Superscript R—resistance.

## Data Availability

The data generated and analyzed during this study are included in this article and its Supplementary Materials. The raw data that support the findings of this study are available on request from the corresponding author.

## Supplementary Materials

The following supporting information can be downloaded at: https://www.mdpi.com/article/10.3390/ijms23169483/s1.
