# Supplementary material for: Interaction between Phage T4 Protein RIII and Host Ribosomal Protein S1 Inhibits Endoribonuclease RegB Activation
Source: Int J Mol Sci. 2022 Aug 22;23(16):9483. doi: 10.3390/ijms23169483 (PMC9409239; doi:10.3390/ijms23169483)
Supplement: Supplementary file 1 [file ijms-23-09483-s001.zip › ijms-1842522-supplementary.pdf]

Supplementary information for:

Article

Interaction between Phage T4 Protein RIII and Host Ribosomal Protein S1 Inhibits Endoribonuclease RegB Activation

Augustinas Juškauskas[1], Aurelija Zajančkauskaitė[1], Rolandas Meškys[1], Marija Ger[2], Algirdas Kaupinis[2], Mindaugas Valius[2] and Lidija Truncaitė[1*]

[1]Department of Molecular Microbiology and Biotechnology, Institute of Biochemistry, Life Sciences Center, Vilnius University, Saulėtekio av. 7, LT-10257 Vilnius, Lithuania.

[2]Proteomics Centre, Institute of Biochemistry, Life Sciences Center, Vilnius University, Saulėtekio av. 7, LT-10257 Vilnius, Lithuania.

*Correspondence: lidija.truncaite@bchi.vu.lt.

Table S1. The list of the oligonucleotide primers used in this study.

No.	Sequence 5′-3′	Used for
1.	CTCTAACGAGAATTTTCATATGATTAAAC	Plasmid 1, 2 construction
2.	ACTAAGGCAGTGGATCCCAATAAATTAC	Plasmid 1, 2 construction
3.	CCTGAAGATTACATATGACTGAATCTTTTGC	Plasmid 4, 6-8 construction
4.	CGAAGGATCCGAGAATTACTCGCCTTTAGCTG	Plasmid 4, 10, 11, 13 construction
5.	TCATTCCGGATAACGTTTAGCGATAG	Plasmid 6 construction
6.	TCACTTGTTGTGGGTTTCCGCGAAC	Plasmid 7 construction
7.	TCATTTCTTGTTCAGAGCAACCCAG	Plasmid 8, 9 construction
8.	GGGCCTGAAACATATGGGCGAAGATC	Plasmid 9, 10 construction
9.	CCTGGGTCTGAAACATATGAAAGCTAACC	Plasmid 11, 12 construction
10.	AAACATATGGCAGAAGATCCGTTCAACAAC	Plasmid 13 construction
11.	GATCTAGACATGACTGAATCTTTTGCTC	Plasmid 14-17 construction
12.	GAGGAGCTCTCGCCTTTAGCTGCTTTG	Plasmid 14 construction
13.	ACCGGTTACGATAGAGCTCTTCTTGTTC	Plasmid 15 construction
14.	TACCTTCAACACGGGAGCTCTTGTTGTG	Plasmid 16 construction
15.	TTGGAGCTCTCCGGATAACGTTTAGC	Plasmid 17 construction
16.	TAATCTCTAACGAGAATCTAGAAATGATTAAAC	Plasmid 18 construction
17.	CAATAGGTACCTTCAGTGTTTACCACAAAGTG	Plasmid 18 construction
18.	AGGATCTAGACATGACTATCAATACAGAAG	Plasmid 19 construction
19.	CATGCGGTACCCATTGAGTTTTAATTACTG	Plasmid 19 construction
20.	CAGTCTAGAGGTGAATAACATGACTATC	Plasmid 20, SD mutation
21.	TGACTCAAAGTGACGACGAA	Plasmid 20, silent mutation of RegB site
22.	GAGTTTCGTCAAATTAACAA	Plasmid 20, silent mutation of RegB site
23.	GCTTCTCGAGTTGAGTTTTAATTACTG	Plasmid 20 construction
24.	TGAGCGGATACATATTTGAATG	Plasmid 21 sequencing
25.	GATTGCACTCAATTTACTTGAG	Plasmid 21 sequencing
26.	GCTCTGGGTCTTAAGCAGCC	Plasmid 21 construction
27.	CAACTTTGTGCAATCTTTGG	Plasmid 21, T4 *rIII* region sequencing
28.	GAACTGTGTGTAGTTCACTCTG	T4 *rIII* region sequencing
29.	GAAGTAGTCCGTAACGCTTTC	Plasmid 22, T4 *regB* region sequencing
30.	GTTATTCTCCTCTTAACTGATAG	Plasmid 22 construction
31.	CTATCAGTTAAGAGGAGAATAACATGACCATGATTACGGATTC	Plasmid 22 construction
32.	GTGCTTTTCTCATGCTTACCTCAGTTTGAGGGGACGACGAC	Plasmid 22 construction
33.	GAGGTAAGCATGAGAAAAGCAC	Plasmid 22 construction
34.	GTAGGCGTGATGCTTTGGTTTAG	Plasmid 22, T4 *regB* sequencing
35.	GCAATGGTAATTAAAATCGTAG	Primer extension
36.	GATGTTTCCAGAGGCAAATAG	Primer extension

The sites for restriction enzymes are underlined.

Table S2. The list of plasmids constructed in this study.

No.	Plasmid	Vector	DNA insert	Primers No.
1.	p16*rIII*His	pET16b, NdeI/BamHI	PCR of *rIII* from T4 wt NdeI/BamHI, N-10 His fusion	1 & 2
2.	p28*rIII*His	pET28a NdeI/BamHI	PCR of *rIII* from T4 wt, NdeI/BamHI, N-6 His fusion	1 & 2
3.	p21d*rIII*His	pET21d, XbaI/BamHI	*rIII* from p28rIIIHis, XbaI/BamHI N-6 His fusion	___
4.	p16*rpsA*His	pET16b NdeI/BamHI	PCR of *rpsA* NdeI/BamHI, N- 10 His fusion	3 & 4
5.	p21*rpsA*	pET21a NdeI/BamHI	*rpsA* gene from p16S1His NdeI/BamHI	___
6.	p21*rpsA*-D1-3	pET21a NdeI/Ecl136II	PCR of *rpsA* 1-828 bp, NdeI, T4 PNK	3 & 5
7.	p21*rpsA*-D1-4	pET21a NdeI/Ecl136II	PCR of *rpsA* 1-1089 bp, NdeI, T4 PNK	3 & 6
8.	p21*rpsA*-D1-5	pET21a NdeI/Ecl136II	PCR of *rpsA* 1-1350 bp, NdeI, T4 PNK	3 & 7
9.	p21*rpsA*-D4-5	pET21a NdeI/Ecl136II	PCR of *rpsA* 785-1350 bp, NdeI, T4 PNK	8 & 7
10.	p21*rpsA*-D4-6	pET21a NdeI/BamHI	PCR of *rpsA* from 785-1674 bp, NdeI, T4 PNK	8 & 4
11.	p21*rpsA*-D5-6	pET21a NdeI/BamHI	PCR of *rpsA* 1093-1674 bp, NdeI, T4 PNK	9 & 4
12.	p21*rpsA*-D5	pET21a NdeI/Ecl136II	PCR of *rpsA* 1093-1350 bp, NdeI, T4 PNK	9 & 7
13.	p21*rpsA*-D6	pET21a NdeI/BamHI	PCR of *rpsA* 1309-1674 bp, NdeI, T4 PNK	10 & 4
14.	pKNT25*rpsA*	pKNT25, XbaI/SacI	PCR of *rpsA* Xba/SacI	11 & 12
15.	pKNT25*rpsA*-D1-5	pKNT25, XbaI/SacI	PCR of *rpsA* 1-1368 bp, XbaI/SacI	11 & 13
16.	pKNT25*rpsA*-D1-4	pKNT25, XbaI/SacI	PCR of *rpsA* 1-1108 bp, XbaI/SacI	11 & 14
17.	pKNT25*rpsA*-D1-3	pKNT25, XbaI/SacI	PCR of *rpsA* 1-836 bp, XbaI/SacI	11 & 15
18.	pUT18*rIII*	pUT18, XbaI/KpnI	PCR of *rIII* from T4 wt, XbaI/KpnI	16 & 17
19.	pUT18*regB*	pUT18, XbaI/KpnI	PCR of *regB* from T4 wt, XbaI/KpnI	18 & 19
20.	p21*regB*SDM2	pET21a XbaI/XhoI	Ligated two PCR of *regB* parts from T4 wt, T4 PNK, XbaI and XhoI, C-terminal 6 His fusion<br>I – PCR of the proximal *regB* part XbaI<br>II – PCR of the distal *regB* part XhoI	I – 20 & 21;<br>II – 22 & 23
21.	pT4*rIII*del	pBSPLO+ HindIII/KpnI	Ligated two DNA fragments:<br>I – PCR with gene *30.9* BstNI (filled in), and<br>II – HindIII-SmaI DNA fragment with gene *31* from plasmid pRA6-1 [83]	I – 26 & 27
22.	pT4*regB*del	pJet1.2/blunt	PCR of three overlapping PCR fragments:<br>I – T4 DNA sequence proximal to *regB* gene;<br>II – *E. coli lacZ alpha* fragment DNA<br>III – T4 DNA sequence distal to *regB* gene.	I – 29 & 30<br>II – 31 & 32<br>III – 33 & 34

A

**Peptide MW, Da**	**Predicted peptide sequence**
1173.66	AFIPGSIVDVR
1282.69	RHEA(DA/W)IT(EI/NK)K
1549.78	KTVELADGVEGYLR

B

>sp|P0AG67|RS1_ECOLI 30S ribosomal protein S1 OS=Escherichia coli (strain K12) OX=83333 GN=rpsA PE=1 SV=1
MTESFAQLFEESLKEIETRPGSIVRGVVVAIDKDVVLVDAGLKSESAIPAEQFKNAQGEL
EIQVGDEVDVALDAVEDGFGETLLSREKAK**RHEAWITLEK**AYEDAETVTGVINGKVKG
GFTVELNGIR**AFLPGSLVDVR**PVRDTLHLEGKELEFKVIKLDQKRNNVVVSRRAVIESEN
SAERDQLLENLQEGMEVKGIVKNLTDYGAFVDLGGVDGLLHITDMAWKRVKHPSEIV
NVGDEITVKVLKFDRERTRVSLGLKQLGEDPWVAIAKRYPEGTKLTGRVTNLTDYGCFV
EIEEGVEGLVHVSEMDWTNKNIHPSKVVNVGDVVEVMVLDIDEERRRISLGLKQCKA
NPWQQFAETHNKGDRVEGKIKSITDFGIFIGLDGGIDGLVHLSDISWNVAGEEAVREYK
KGDEIAAVVLQVDAERERISLGVKQLAEDPFNNWVALNKKGAIVTGKVTAVDAKG**ATV**
ELADGVEGYLRASEASRDRVEDATLVLSVGDEVEAKFTGVDRKNRAISLSVRAKDEADE
KDAIATVNKQEDANFSNNAMAEAFKAAKGE

Figure S1. Identification of *E. coli* ribosomal protein S1. **(A)** The sequences of peptides predicted in *de novo* sequencing analysis. **(B)** The sequence of *E. coli* ribosomal protein S1 with the peptides identified marked in bold. The non-matching amino acids are shown in red. The L and I have equal molecular mass, so should be interpreted equally.

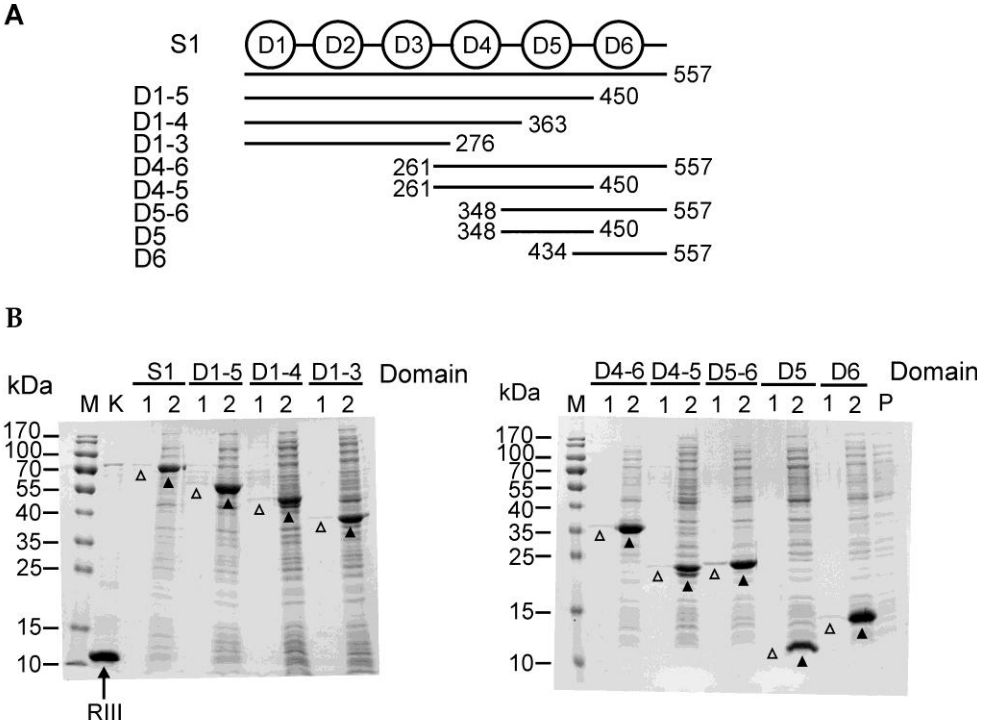

Figure S2. The negative controls of the pull-down assays performed in this study. These control assays were performed without immobilization of the recombinant His-tagged RIII protein. **(A)** The schematic representation of domains of the intact ribosomal protein S1 (six domains) and its truncated variants used for analysis. The numbers next to the lines mean the amino acid positions of S1. **(B)** The images of 14% SDS-PAGE of negative controls of the pull-down assays. The lysates of induced *E. coli* BL21 (DE3) cells (Novagen, Madison, WI, USA) carrying pET21d (Novagen, Madison, WI, USA) plasmid were loaded on a blank nickel-charged His-affinity agarose, washed, and then mixed with cell lysates containing either induced intact S1 or its truncated variants (indicated on the top of the gel). Lanes: M – PageRuler™ Prestained Protein Ladder (Thermo Scientific, Vilnius, Lithuania); K – the sample of the purified His-tagged RIII protein, which is also shown by black arrow; 1 – non-specifically bound prey proteins; 2 – the samples of induced *E. coli* BL21 (DE3) cell lysates with induced prey proteins. P – the sample of *E. coli* BL21 (DE3) cell lysate carrying plasmid vector pET21d. The positions of S1 and its truncated variants are marked with black arrowheads; open arrowheads indicate the trace amounts of the respective S1 variants nonspecifically bound to the agarose.

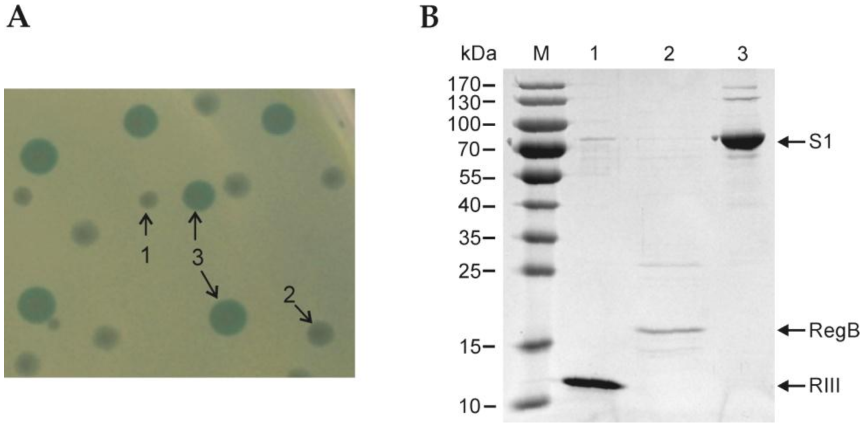

Figure S3. The plaques of phages and the purified recombinant proteins used for *in vitro* RegB cleavage assays. **(A)** The plaques of phages T4 wt (1), T4 Δ*rIII* (2) and T4 Δ*rIII*Δ*regB* (3) on the lawns of *E. coli* DH10B (Thermo Scientific, Vilnius, Lithuania) in the presence of X-gal (200 µg ml^{-1}) and IPTG (0.5 mM). **(B)** The image of 14% SDS-PAGE of the samples of purified His-tagged recombinant proteins used for *in vitro* RegB cleavage assays. Lanes: M – PageRuler™ Prestained Protein Ladder (Thermo Scientific, Vilnius, Lithuania); 1 – RIII; 2 – RegB and 3 – S1 that are also marked by arrows from the right side.

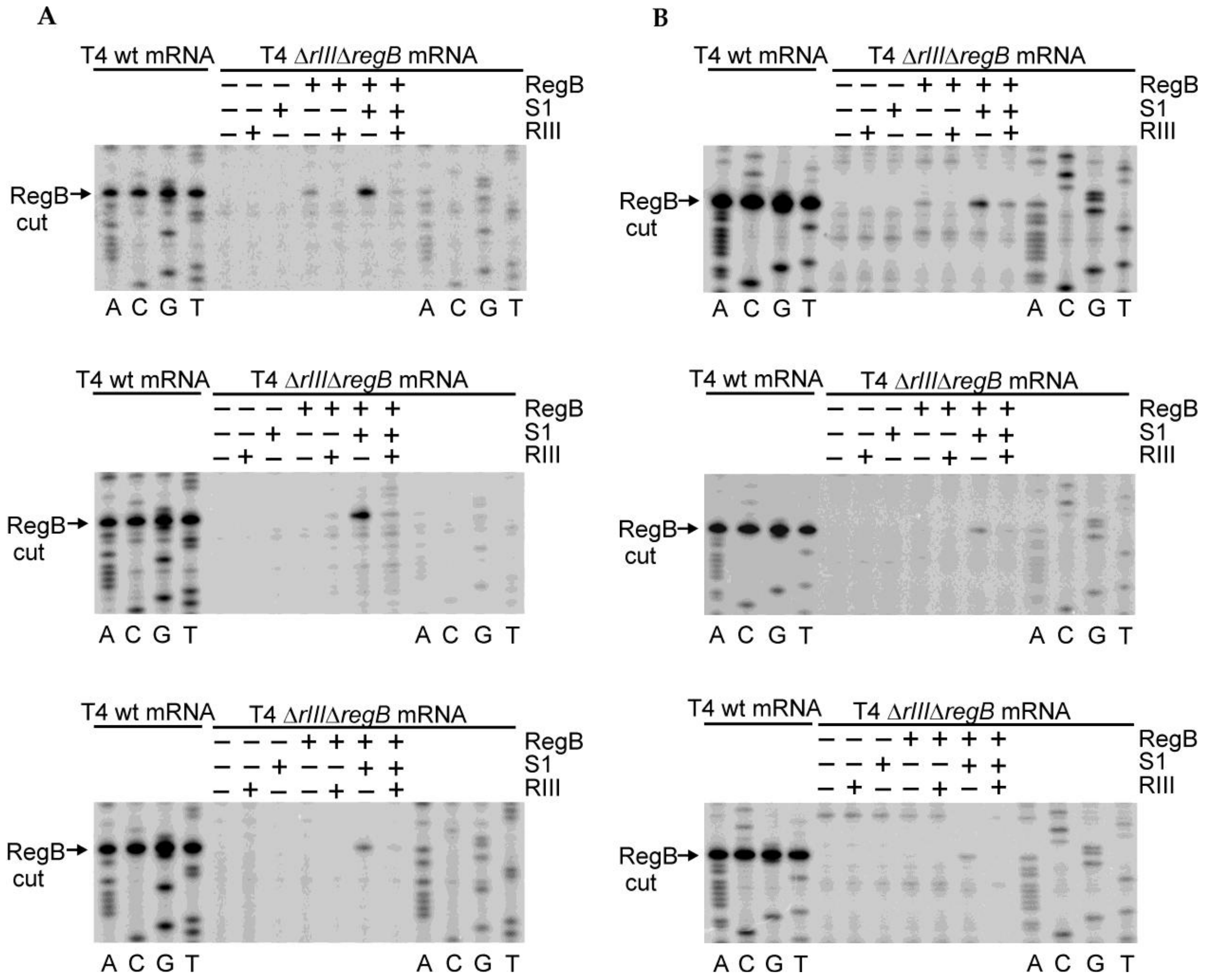

Figure S4. Primer extension analysis of T4 early mRNAs *in vitro* exposed to diverse sets of recombinant proteins. **(A)** The images of the gels from three independent experiments using 5′-^{32}P-labelled primer No 35. complementary to the gene *motA* mRNA. **(B)** The images of gels from three independent experiments using 5′-^{32}P-labelled primer No. 36 complementary to the gene *30.7* mRNA. Total RNA was isolated from the *E. coli* cells 2 min postinfection with either phage T4 wt or T4 Δ*rIII*Δ*regB* at 37 °C. The aliquots of RNA were subjected either to the Sanger dideoxy sequencing or to the primer extension analysis. The latter analysis was performed following exposure of RNA to different sets of the His-tagged recombinant proteins specified by + or – on the top of the figure.
